# 1-(2-Chloro­benz­yl)-3-methyl-2,6-diphenyl­piperidine

**DOI:** 10.1107/S1600536812029200

**Published:** 2012-06-30

**Authors:** Chennan Ramalingan, Seik Weng Ng, Edward R. T. Tiekink

**Affiliations:** a Centre for Nanotechnology, Department of Chemistry, Kalasalingam University, Krishnankoil 626 126, Tamilnadu, India; b Department of Chemistry, University of Malaya, 50603 Kuala Lumpur, Malaysia; c Chemistry Department and Faculty of Science, King Abdulaziz University, PO Box 80203 Jeddah, Saudi Arabia

## Abstract

In the title compound, C_25_H_26_ClN, the piperidine ring has a chair conformation with all ring substituents in equatorial positions. The dihedral angle formed between the chloro­benzene ring and the flanking phenyl rings are 74.91 (18) and 47.86 (17)°. The chloro substituent is *anti* to the piperidine N atom. In the crystal, centrosymmetrically related mol­ecules aggregate *via* π–π inter­actions occurring between chloro­benzene rings [centroid–centroid distance = 3.778 (2) Å] and these are linked into linear supra­molecular chains along the *a* axis by C—H⋯π inter­actions occurring between the phenyl rings.

## Related literature

For the biological activity of piperidine derivatives, see: Ramalingan *et al.* (2004 ▶); Ramachandran *et al.* (2011 ▶). For a related structure, see: Ramalingan *et al.* (2012 ▶).
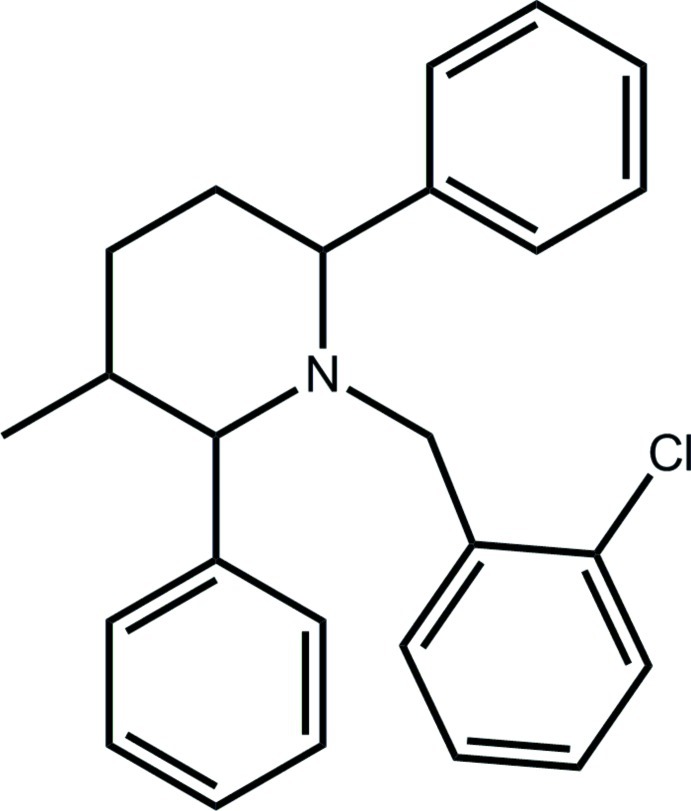


## Experimental

### 

#### Crystal data

C_25_H_26_ClN
*M* *_r_* = 375.92
Triclinic, 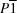

*a* = 10.0878 (7) Å
*b* = 10.2837 (5) Å
*c* = 11.3583 (7) Å
α = 94.150 (5)°
β = 107.713 (6)°
γ = 111.065 (5)°
*V* = 1025.32 (11) Å^3^
*Z* = 2
Mo *K*α radiation
μ = 0.20 mm^−1^
*T* = 100 K
0.25 × 0.15 × 0.03 mm

#### Data collection

Agilent SuperNova Dual diffractometer with an Atlas detector
Absorption correction: multi-scan (*CrysAlis PRO*; Agilent, 2012 ▶) *T*_min_ = 0.495, *T*_max_ = 1.000
7033 measured reflections
4678 independent reflections
2850 reflections with *I* > 2σ(*I*)
*R*_int_ = 0.034

#### Refinement

*R*[*F* ^2^ > 2σ(*F* ^2^)] = 0.074
*wR*(*F* ^2^) = 0.185
*S* = 1.03
4678 reflections
244 parameters
12 restraints
H-atom parameters constrained
Δρ_max_ = 0.72 e Å^−3^
Δρ_min_ = −0.47 e Å^−3^

### 

Data collection: *CrysAlis PRO* (Agilent, 2012 ▶); cell refinement: *CrysAlis PRO*; data reduction: *CrysAlis PRO*; program(s) used to solve structure: *SHELXS97* (Sheldrick, 2008 ▶); program(s) used to refine structure: *SHELXL97* (Sheldrick, 2008 ▶); molecular graphics: *ORTEP-3 for Windows* (Farrugia, 1997 ▶) and *DIAMOND* (Brandenburg, 2006 ▶); software used to prepare material for publication: *publCIF* (Westrip, 2010 ▶).

## Supplementary Material

Crystal structure: contains datablock(s) I, global. DOI: 10.1107/S1600536812029200/hb6872sup1.cif

Structure factors: contains datablock(s) I. DOI: 10.1107/S1600536812029200/hb6872Isup2.hkl

Supplementary material file. DOI: 10.1107/S1600536812029200/hb6872Isup3.cml

Additional supplementary materials:  crystallographic information; 3D view; checkCIF report

## Figures and Tables

**Table 1:** Hydrogen-bond geometry (Å, °) *Cg*1 is the centroid of the C20–C25 ring.

*D*—H⋯*A*	*D*—H	H⋯*A*	*D*⋯*A*	*D*—H⋯*A*
C17—H17⋯*Cg*1^i^	0.95	2.83	3.692 (4)	151

Symmetry code: (i) 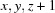.

**Fig. 1.**
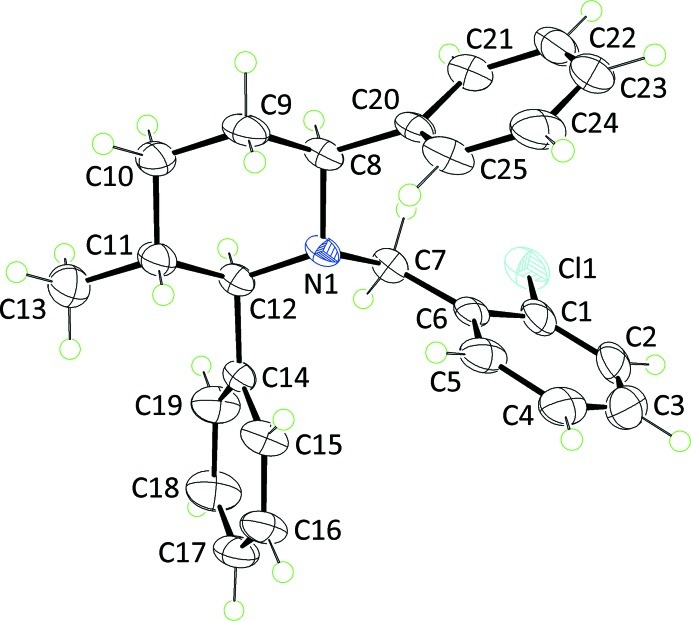
The molecular structure of (I) showing displacement ellipsoids at the 50% probability level.

**Fig. 2.**
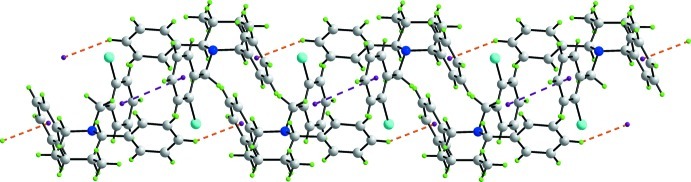
A view of the supramolecular chain in (I) sustained by C—H···π and π—π interactions which are shown as orange and purple dashed lines, respectively

**Fig. 3.**
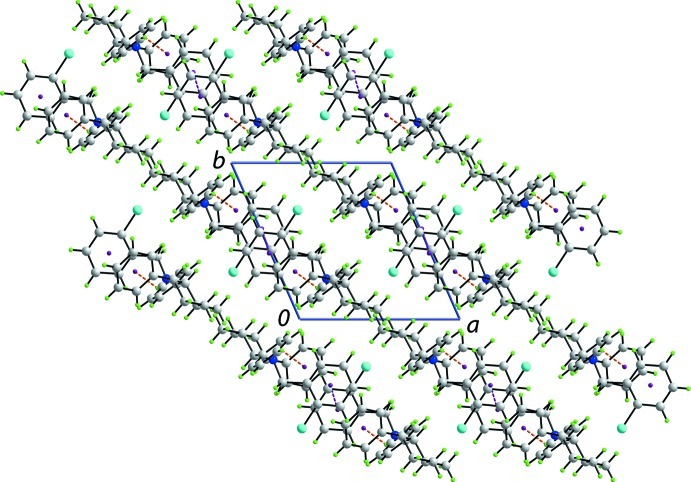
A view in projection down the a axis of the unit-cell contents for (I). The C—H···π and π—π interactions are shown as orange and purple dashed lines, respectively.

## supplementary crystallographic information

### Comment

Piperidine derivatives are an important class of heterocyclic compounds with potential applications in medicinal chemistry as these can be frequently recognized in the structures of various synthetic targets as well as naturally occurring alkaloids (Ramalingan *et al.*, 2004; Ramachandran *et al.*, 2011). The title compound, (I), was designed and synthesized to evaluate its biological properties. The crystal structure determination was undertaken in order to establish conformational details.

In (I), Fig. 1, the piperidine ring has a chair conformation and all ring-substituents occupy equatorial positions. The dihedral angle formed between the C1–C6 benzene ring and the flanking C14–C19 and C20–C25 phenyl rings are 74.91 (18) and 47.86 (17)°, respectively; the dihedral angle between the phenyl rings is 58.93 (18)°. In a comparable molecule, having an extra C-bound methyl group (Ramalingan *et al.*, 2012), these substituents were found to occupy the same positions. The chloro substituent is *anti* to the piperidine-N atom.

In the crystal packing, centrosymmetrically related molecules aggregate *via*π—π interactions occurring between chlorobenzene rings [inter-centroid distance = 3.778 (2) Å for symmetry operation 2 - *x*, 1 - *y*, 1 - *z*]. These are linked into linear supramolecular chains along the *a* axis by C—H···π interactions whereby a phenyl-H17 atom associates with the C20—C25 ring, Fig. 2 and Table 1. Chains aggregate into layers in the *ab* plane without specific intermolecular interactions between them, Fig. 3.

### Experimental

A starting material, 3-methyl-2,6-diphenylpiperidine, was synthesized from benzaldehyde, 2-butanone and ammonium acetate through a Mannich-type reaction (for a typical synthesis, see Ramalingan *et al.* (2004)) followed by standard Wolff-Kishner reduction using hydrazine hydrate in diethylene glycol. 1-(2-Chlorobenzyl)-3-methyl-2,6-diphenylpiperidine was then synthesized as follows. To a DMF solution (15 ml) of 3-methyl-2,6-diphenylpiperidine (1.26 g, 0.005 mol) was added potassium *tert*-butoxide (0.67 g, 0.006 mol). The mixture was stirred for 30 minutes and 2-chlorobenzyl bromide (0.78 ml, 0.006 mol) was added drop-wise. Stirring was continued overnight before aqueous work-up. Extraction with diethyl ether followed by column chromatography separation using n-hexane/ethyl acetate (100:4) as an eluent eventually provided the pure title compound as a white solid. Re-crystallization was performed by slow evaporation of its ethanolic solution which afforded colourless plates. *M*.pt: 352–353 K. Yield: 83%.

### Refinement

Carbon-bound H-atoms were placed in calculated positions [C—H = 0.95–1.00 Å, *U*_iso_(H) = 1.2–1.5*U*_eq_(C)] and were included in the refinement in the riding model approximation. The anisotropic displacement parameters for the C3 and C4 atoms were constrained to be nearly isotropic.

### Crystal data

**Table:**

C_25_H_26_ClN	*Z* = 2
*M**_r_* = 375.92	*F*(000) = 400
Triclinic, *P*1	*D*_x_ = 1.218 Mg m^−^^3^
Hall symbol: -P 1	Mo *K*α radiation, λ = 0.71073 Å
*a* = 10.0878 (7) Å	Cell parameters from 1908 reflections
*b* = 10.2837 (5) Å	θ = 2.4–27.5°
*c* = 11.3583 (7) Å	µ = 0.20 mm^−^^1^
α = 94.150 (5)°	*T* = 100 K
β = 107.713 (6)°	Plate, colourless
γ = 111.065 (5)°	0.25 × 0.15 × 0.03 mm
*V* = 1025.32 (11) Å^3^	

### Data collection

**Table:**

Agilent SuperNova Dual diffractometer with an Atlas detector	4678 independent reflections
Radiation source: SuperNova (Mo) X-ray Source	2850 reflections with *I* > 2σ(*I*)
Mirror monochromator	*R*_int_ = 0.034
Detector resolution: 10.4041 pixels mm^-1^	θ_max_ = 27.6°, θ_min_ = 2.4°
ω scan	*h* = −13→9
Absorption correction: multi-scan (*CrysAlis PRO*; Agilent, 2012)	*k* = −13→13
*T*_min_ = 0.495, *T*_max_ = 1.000	*l* = −13→14
7033 measured reflections	

### Refinement

**Table:**

Refinement on *F*^2^	Primary atom site location: structure-invariant direct methods
Least-squares matrix: full	Secondary atom site location: difference Fourier map
*R*[*F*^2^ > 2σ(*F*^2^)] = 0.074	Hydrogen site location: inferred from neighbouring sites
*wR*(*F*^2^) = 0.185	H-atom parameters constrained
*S* = 1.03	*w* = 1/[σ^2^(*F*_o_^2^) + (0.0541*P*)^2^ + 1.0947*P*] where *P* = (*F*_o_^2^ + 2*F*_c_^2^)/3
4678 reflections	(Δ/σ)_max_ < 0.001
244 parameters	Δρ_max_ = 0.72 e Å^−^^3^
12 restraints	Δρ_min_ = −0.47 e Å^−^^3^

### Special details

**Table:**

Geometry. All e.s.d.'s (except the e.s.d. in the dihedral angle between two l.s. planes) are estimated using the full covariance matrix. The cell e.s.d.'s are taken into account individually in the estimation of e.s.d.'s in distances, angles and torsion angles; correlations between e.s.d.'s in cell parameters are only used when they are defined by crystal symmetry. An approximate (isotropic) treatment of cell e.s.d.'s is used for estimating e.s.d.'s involving l.s. planes.
Refinement. Refinement of *F*^2^ against ALL reflections. The weighted *R*-factor *wR* and goodness of fit *S* are based on *F*^2^, conventional *R*-factors *R* are based on *F*, with *F* set to zero for negative *F*^2^. The threshold expression of *F*^2^ > σ(*F*^2^) is used only for calculating *R*-factors(gt) *etc*. and is not relevant to the choice of reflections for refinement. *R*-factors based on *F*^2^ are statistically about twice as large as those based on *F*, and *R*- factors based on ALL data will be even larger.

### Fractional atomic coordinates and isotropic or equivalent isotropic displacement parameters (Å^2^)

**Table:**

	*x*	*y*	*z*	*U*_iso_*/*U*_eq_	
Cl1	0.71459 (12)	0.30264 (9)	0.23928 (9)	0.0512 (3)	
C1	0.8802 (4)	0.4580 (4)	0.3038 (3)	0.0395 (8)	
C2	1.0149 (5)	0.4438 (5)	0.3581 (3)	0.0522 (10)	
H2	1.0170	0.3523	0.3591	0.063*	
C3	1.1464 (5)	0.5651 (6)	0.4110 (4)	0.0622 (12)	
H3	1.2396	0.5560	0.4494	0.075*	
C4	1.1470 (4)	0.7006 (5)	0.4099 (3)	0.0482 (10)	
H4	1.2386	0.7836	0.4462	0.058*	
C5	1.0061 (4)	0.7099 (4)	0.3528 (3)	0.0395 (8)	
H5	1.0039	0.8013	0.3505	0.047*	
C6	0.8716 (3)	0.5909 (3)	0.3004 (3)	0.0284 (7)	
N1	0.7224 (3)	0.7408 (2)	0.2310 (2)	0.0260 (6)	
C7	0.7188 (3)	0.5974 (3)	0.2470 (3)	0.0287 (7)	
H7A	0.6623	0.5315	0.1637	0.034*	
H7B	0.6614	0.5625	0.3032	0.034*	
C8	0.6816 (4)	0.7457 (3)	0.0938 (3)	0.0308 (7)	
H8	0.5797	0.6671	0.0471	0.037*	
C9	0.6704 (4)	0.8861 (3)	0.0710 (3)	0.0390 (8)	
H9A	0.6383	0.8849	−0.0207	0.047*	
H9B	0.7715	0.9652	0.1128	0.047*	
C10	0.5567 (4)	0.9107 (4)	0.1226 (3)	0.0386 (8)	
H10A	0.5526	1.0033	0.1086	0.046*	
H10B	0.4542	0.8349	0.0773	0.046*	
C11	0.6033 (4)	0.9101 (3)	0.2629 (3)	0.0367 (8)	
H11	0.7050	0.9898	0.3071	0.044*	
C12	0.6174 (4)	0.7696 (3)	0.2865 (3)	0.0289 (7)	
H12	0.5145	0.6903	0.2462	0.035*	
C13	0.4882 (5)	0.9353 (4)	0.3160 (3)	0.0508 (10)	
H13A	0.4819	1.0256	0.2998	0.076*	
H13B	0.5220	0.9390	0.4072	0.076*	
H13C	0.3880	0.8573	0.2747	0.076*	
C14	0.6717 (3)	0.7731 (3)	0.4273 (3)	0.0257 (6)	
C15	0.8186 (4)	0.8583 (3)	0.5027 (3)	0.0373 (8)	
H15	0.8873	0.9119	0.4653	0.045*	
C16	0.8687 (4)	0.8678 (4)	0.6320 (3)	0.0425 (9)	
H16	0.9714	0.9261	0.6822	0.051*	
C17	0.7699 (4)	0.7930 (4)	0.6883 (3)	0.0430 (9)	
H17	0.8030	0.8017	0.7773	0.052*	
C18	0.6248 (5)	0.7071 (4)	0.6151 (3)	0.0537 (11)	
H18	0.5566	0.6544	0.6532	0.064*	
C19	0.5744 (4)	0.6950 (4)	0.4837 (3)	0.0443 (9)	
H19	0.4730	0.6330	0.4333	0.053*	
C20	0.7954 (4)	0.7211 (3)	0.0442 (3)	0.0299 (7)	
C21	0.7525 (4)	0.5955 (3)	−0.0406 (3)	0.0367 (8)	
H21	0.6511	0.5262	−0.0672	0.044*	
C22	0.8556 (5)	0.5702 (4)	−0.0870 (3)	0.0459 (9)	
H22	0.8247	0.4838	−0.1441	0.055*	
C23	1.0022 (5)	0.6704 (4)	−0.0500 (3)	0.0488 (10)	
H23	1.0730	0.6536	−0.0814	0.059*	
C24	1.0459 (4)	0.7955 (4)	0.0331 (3)	0.0488 (10)	
H24	1.1470	0.8651	0.0587	0.059*	
C25	0.9436 (4)	0.8202 (4)	0.0791 (3)	0.0412 (9)	
H25	0.9754	0.9071	0.1360	0.049*	

### Atomic displacement parameters (Å^2^)

**Table:**

	*U*^11^	*U*^22^	*U*^33^	*U*^12^	*U*^13^	*U*^23^
Cl1	0.0784 (7)	0.0327 (4)	0.0474 (5)	0.0239 (5)	0.0270 (5)	0.0073 (4)
C1	0.053 (2)	0.053 (2)	0.0308 (16)	0.0303 (18)	0.0270 (16)	0.0130 (15)
C2	0.069 (3)	0.083 (3)	0.039 (2)	0.054 (3)	0.035 (2)	0.023 (2)
C3	0.047 (2)	0.119 (4)	0.042 (2)	0.047 (3)	0.0269 (19)	0.024 (2)
C4	0.0341 (19)	0.082 (3)	0.0307 (18)	0.0177 (19)	0.0204 (15)	0.0145 (18)
C5	0.0325 (18)	0.052 (2)	0.0270 (16)	0.0060 (16)	0.0147 (15)	0.0051 (15)
C6	0.0312 (16)	0.0384 (17)	0.0195 (14)	0.0126 (14)	0.0158 (13)	0.0069 (12)
N1	0.0342 (14)	0.0246 (12)	0.0213 (12)	0.0102 (11)	0.0147 (11)	0.0053 (10)
C7	0.0316 (17)	0.0235 (14)	0.0290 (15)	0.0065 (13)	0.0140 (13)	0.0037 (12)
C8	0.0398 (18)	0.0295 (15)	0.0222 (14)	0.0105 (14)	0.0145 (14)	0.0025 (12)
C9	0.062 (2)	0.0353 (17)	0.0218 (15)	0.0190 (17)	0.0173 (16)	0.0092 (13)
C10	0.060 (2)	0.0386 (18)	0.0266 (16)	0.0283 (17)	0.0165 (16)	0.0123 (14)
C11	0.055 (2)	0.0365 (17)	0.0253 (15)	0.0243 (16)	0.0160 (15)	0.0082 (13)
C12	0.0338 (17)	0.0297 (15)	0.0243 (15)	0.0112 (13)	0.0136 (13)	0.0049 (12)
C13	0.070 (3)	0.058 (2)	0.042 (2)	0.038 (2)	0.026 (2)	0.0164 (18)
C14	0.0306 (16)	0.0254 (14)	0.0227 (14)	0.0107 (13)	0.0124 (13)	0.0040 (12)
C15	0.0389 (19)	0.0342 (17)	0.0284 (16)	0.0018 (15)	0.0147 (15)	0.0005 (14)
C16	0.042 (2)	0.046 (2)	0.0263 (16)	0.0095 (17)	0.0070 (16)	−0.0050 (15)
C17	0.062 (2)	0.0429 (19)	0.0227 (15)	0.0210 (18)	0.0136 (17)	0.0072 (14)
C18	0.059 (2)	0.060 (2)	0.038 (2)	0.009 (2)	0.0293 (19)	0.0197 (18)
C19	0.0365 (19)	0.050 (2)	0.0355 (18)	0.0038 (17)	0.0154 (16)	0.0078 (16)
C20	0.0413 (18)	0.0320 (16)	0.0189 (13)	0.0133 (14)	0.0157 (13)	0.0065 (12)
C21	0.045 (2)	0.0311 (16)	0.0293 (16)	0.0087 (15)	0.0151 (15)	0.0039 (13)
C22	0.072 (3)	0.046 (2)	0.0320 (18)	0.031 (2)	0.0261 (19)	0.0075 (16)
C23	0.059 (2)	0.076 (3)	0.0330 (18)	0.037 (2)	0.0304 (18)	0.0223 (19)
C24	0.045 (2)	0.065 (2)	0.0323 (18)	0.0119 (19)	0.0200 (17)	0.0140 (18)
C25	0.047 (2)	0.0412 (19)	0.0279 (16)	0.0066 (17)	0.0176 (16)	0.0005 (14)

### Geometric parameters (Å, º)

**Table:**

Cl1—C1	1.746 (4)	C11—H11	1.0000
C1—C2	1.376 (5)	C12—C14	1.518 (4)
C1—C6	1.402 (5)	C12—H12	1.0000
C2—C3	1.376 (6)	C13—H13A	0.9800
C2—H2	0.9500	C13—H13B	0.9800
C3—C4	1.393 (6)	C13—H13C	0.9800
C3—H3	0.9500	C14—C15	1.372 (4)
C4—C5	1.413 (5)	C14—C19	1.381 (4)
C4—H4	0.9500	C15—C16	1.384 (4)
C5—C6	1.379 (4)	C15—H15	0.9500
C5—H5	0.9500	C16—C17	1.379 (5)
C6—C7	1.504 (4)	C16—H16	0.9500
N1—C12	1.487 (4)	C17—C18	1.355 (5)
N1—C7	1.488 (4)	C17—H17	0.9500
N1—C8	1.496 (4)	C18—C19	1.402 (5)
C7—H7A	0.9900	C18—H18	0.9500
C7—H7B	0.9900	C19—H19	0.9500
C8—C20	1.511 (4)	C20—C25	1.384 (4)
C8—C9	1.523 (4)	C20—C21	1.395 (4)
C8—H8	1.0000	C21—C22	1.391 (5)
C9—C10	1.523 (5)	C21—H21	0.9500
C9—H9A	0.9900	C22—C23	1.374 (5)
C9—H9B	0.9900	C22—H22	0.9500
C10—C11	1.520 (4)	C23—C24	1.380 (5)
C10—H10A	0.9900	C23—H23	0.9500
C10—H10B	0.9900	C24—C25	1.378 (5)
C11—C12	1.536 (4)	C24—H24	0.9500
C11—C13	1.549 (5)	C25—H25	0.9500
			
C2—C1—C6	122.7 (4)	C13—C11—H11	108.3
C2—C1—Cl1	117.7 (3)	N1—C12—C14	109.9 (2)
C6—C1—Cl1	119.6 (3)	N1—C12—C11	112.0 (2)
C1—C2—C3	118.6 (4)	C14—C12—C11	109.8 (2)
C1—C2—H2	120.7	N1—C12—H12	108.3
C3—C2—H2	120.7	C14—C12—H12	108.3
C2—C3—C4	122.0 (4)	C11—C12—H12	108.3
C2—C3—H3	119.0	C11—C13—H13A	109.5
C4—C3—H3	119.0	C11—C13—H13B	109.5
C3—C4—C5	117.4 (4)	H13A—C13—H13B	109.5
C3—C4—H4	121.3	C11—C13—H13C	109.5
C5—C4—H4	121.3	H13A—C13—H13C	109.5
C6—C5—C4	122.2 (4)	H13B—C13—H13C	109.5
C6—C5—H5	118.9	C15—C14—C19	118.2 (3)
C4—C5—H5	118.9	C15—C14—C12	120.4 (3)
C5—C6—C1	117.1 (3)	C19—C14—C12	121.4 (3)
C5—C6—C7	123.5 (3)	C14—C15—C16	121.3 (3)
C1—C6—C7	119.4 (3)	C14—C15—H15	119.3
C12—N1—C7	108.8 (2)	C16—C15—H15	119.3
C12—N1—C8	112.7 (2)	C17—C16—C15	120.2 (3)
C7—N1—C8	108.9 (2)	C17—C16—H16	119.9
N1—C7—C6	115.3 (2)	C15—C16—H16	119.9
N1—C7—H7A	108.4	C18—C17—C16	119.3 (3)
C6—C7—H7A	108.4	C18—C17—H17	120.4
N1—C7—H7B	108.4	C16—C17—H17	120.4
C6—C7—H7B	108.4	C17—C18—C19	120.7 (3)
H7A—C7—H7B	107.5	C17—C18—H18	119.6
N1—C8—C20	110.2 (3)	C19—C18—H18	119.6
N1—C8—C9	111.0 (2)	C14—C19—C18	120.2 (3)
C20—C8—C9	111.2 (3)	C14—C19—H19	119.9
N1—C8—H8	108.1	C18—C19—H19	119.9
C20—C8—H8	108.1	C25—C20—C21	117.6 (3)
C9—C8—H8	108.1	C25—C20—C8	122.3 (3)
C8—C9—C10	110.8 (3)	C21—C20—C8	120.1 (3)
C8—C9—H9A	109.5	C22—C21—C20	121.1 (3)
C10—C9—H9A	109.5	C22—C21—H21	119.4
C8—C9—H9B	109.5	C20—C21—H21	119.4
C10—C9—H9B	109.5	C23—C22—C21	119.9 (3)
H9A—C9—H9B	108.1	C23—C22—H22	120.1
C11—C10—C9	110.0 (3)	C21—C22—H22	120.1
C11—C10—H10A	109.7	C22—C23—C24	119.6 (4)
C9—C10—H10A	109.7	C22—C23—H23	120.2
C11—C10—H10B	109.7	C24—C23—H23	120.2
C9—C10—H10B	109.7	C25—C24—C23	120.4 (3)
H10A—C10—H10B	108.2	C25—C24—H24	119.8
C10—C11—C12	110.2 (2)	C23—C24—H24	119.8
C10—C11—C13	110.3 (3)	C24—C25—C20	121.4 (3)
C12—C11—C13	111.4 (3)	C24—C25—H25	119.3
C10—C11—H11	108.3	C20—C25—H25	119.3
C12—C11—H11	108.3		
			
C6—C1—C2—C3	−0.2 (5)	C10—C11—C12—N1	54.7 (4)
Cl1—C1—C2—C3	−179.1 (3)	C13—C11—C12—N1	177.5 (3)
C1—C2—C3—C4	−0.6 (5)	C10—C11—C12—C14	177.1 (3)
C2—C3—C4—C5	0.5 (5)	C13—C11—C12—C14	−60.1 (3)
C3—C4—C5—C6	0.5 (5)	N1—C12—C14—C15	52.4 (4)
C4—C5—C6—C1	−1.2 (4)	C11—C12—C14—C15	−71.3 (4)
C4—C5—C6—C7	175.6 (3)	N1—C12—C14—C19	−129.9 (3)
C2—C1—C6—C5	1.0 (4)	C11—C12—C14—C19	106.5 (4)
Cl1—C1—C6—C5	179.9 (2)	C19—C14—C15—C16	−0.7 (5)
C2—C1—C6—C7	−175.9 (3)	C12—C14—C15—C16	177.1 (3)
Cl1—C1—C6—C7	3.0 (4)	C14—C15—C16—C17	−1.3 (6)
C12—N1—C7—C6	−131.3 (2)	C15—C16—C17—C18	2.0 (6)
C8—N1—C7—C6	105.4 (3)	C16—C17—C18—C19	−0.8 (6)
C5—C6—C7—N1	11.4 (4)	C15—C14—C19—C18	1.9 (5)
C1—C6—C7—N1	−171.9 (3)	C12—C14—C19—C18	−175.9 (3)
C12—N1—C8—C20	177.9 (2)	C17—C18—C19—C14	−1.2 (6)
C7—N1—C8—C20	−61.2 (3)	N1—C8—C20—C25	−69.8 (4)
C12—N1—C8—C9	54.3 (3)	C9—C8—C20—C25	53.7 (4)
C7—N1—C8—C9	175.2 (3)	N1—C8—C20—C21	110.8 (3)
N1—C8—C9—C10	−56.3 (3)	C9—C8—C20—C21	−125.7 (3)
C20—C8—C9—C10	−179.4 (2)	C25—C20—C21—C22	0.9 (5)
C8—C9—C10—C11	58.1 (3)	C8—C20—C21—C22	−179.6 (3)
C9—C10—C11—C12	−56.7 (4)	C20—C21—C22—C23	−0.6 (5)
C9—C10—C11—C13	179.9 (3)	C21—C22—C23—C24	0.1 (5)
C7—N1—C12—C14	62.9 (3)	C22—C23—C24—C25	0.1 (5)
C8—N1—C12—C14	−176.1 (2)	C23—C24—C25—C20	0.2 (5)
C7—N1—C12—C11	−174.7 (2)	C21—C20—C25—C24	−0.8 (5)
C8—N1—C12—C11	−53.7 (3)	C8—C20—C25—C24	179.8 (3)

### Hydrogen-bond geometry (Å, º)

Cg1 is the centroid of the C20–C25 ring.

**Table:**

*D*—H···*A*	*D*—H	H···*A*	*D*···*A*	*D*—H···*A*
C17—H17···*Cg*1^i^	0.95	2.83	3.692 (4)	151

Symmetry code: (i) *x*, *y*, *z*+1.
